# Case of Spontaneous Dislocation and Anchylosis of the First and Second Cervical Vertebræ

**Published:** 1842-04-16

**Authors:** 


*Case of Spontaneous Dislocation and Anchylosis of the First and Second Cervical Verterbræ.*—The patient in this case was a shoemaker, and at the time of his death was thirty years of age. He was a muscular man, of moderate stature, and from his youth had had stiff neck; he always carried his head towards his left shoulder, and moved it only with the trunk. From the account of his friends, it appeared that when about nine years of age, he had an obscure complaint in his throat and neck, and that for a long time afterwards he had been obliged to turn his head with great caution. The manner of his death is thus described:—"He had been drinking almost all day, and towards evening he laid his head on the table of a beer-shop and fell asleep. He continued in that posture about an hour, when waking suddenly, he made an effort to raise himself—staggered across the room, and fell down without a groan or struggle." On examination, the brain was found intensely gorged with venous blood, and a small coagulum was discovered on the lower and outer surface of the right middle lobe. These were the only changes of moment observed in the brain. The atlas and vertebra dentata were firmly anchylosed together, a degree of displacement having previously taken place, of which the extent could hardly have been adequately estimated without an inspection of the preparation which accompanied the paper. It must suffice to state here that the dimensions of the space, as given by the author, occupied by the medulla oblongata, were as follows:

From side to side, 0.9 of an inch.

From before backwards at the widest part, 0.3 of an inch.

From the right surface of the odontoid process to the opposite surface of the atlas, 0.1 of an inch.

"This frightful displacement," says the author, "was doubtless occasioned by ulceration of the transverse ligament, and it is very probable that life might have been preserved for many years longer but for the indulgence of habits which added vascular turgescence to the risk arising from a permanent constricted medulla."

*Provin. Med. and Surg. Journ.*, Feb. 12th.

